# Readiness of health professionals for telemedicine implementation: multi-centered cross-sectional study in public hospitals, South Ethiopia

**DOI:** 10.3389/fdgth.2025.1554199

**Published:** 2025-07-02

**Authors:** Yenus Ibrahim, Sewunet Sako, Abdurehman Ayele, Mustefa Glagn

**Affiliations:** ^1^ Unit of Health Service Management, School of Public Health, College of Medicine and Health Sciences, Arba Minch University, Arba Minch, Ethiopia; ^2^ Department of Health Informatics, School of Public Health, College of Medicine and Health Sciences, Arba Minch University, Arba Minch, Ethiopia; ^3^ Department of Comprehensive Nursing, College of Medicine and Health Sciences, Arba Minch University, Arba Minch, Ethiopia

**Keywords:** readiness, telemedicine, health professionals, associated factors, South Ethiopia

## Abstract

**Background:**

The application of telemedicine significantly improves healthcare access and quality. However, its implementation is limited by healthcare professionals’ readiness, particularly in low-income countries like Ethiopia. This study aimed to assess healthcare professionals’ readiness and identify factors influencing it.

**Methods:**

An Institution-based cross-sectional study was conducted among 417 healthcare professionals in six public hospitals in the Gamo zone from July to August 2024. A simple random sampling technique was employed to select the study participants. The data were collected using a Kobo self-administered structured questionnaire and were exported to SPSSv.26 for analysis. Variables with *p*-values ≤ 0.25 were candidates for multivariable logistic regressions and significance was determined at *p*-values of <0.05 at a 95% confidence level.

**Result:**

Healthcare professionals’ readiness was found to be 68.8%(95%CI: 64.5%–72.8%). Computer access [AOR: 2.23(95% CI: 1.26–3.93)], having adequate knowledge [AOR: 2.01(95% CI: 1.29–3.41)], having a favorable attitude [AOR: 1.96(95% CI 1.23–3.131)], and being female [AOR: 1.782(95%CI: 1.119–2.840)] were significantly associated with readiness of health professionals.

**Conclusion:**

Two-thirds of health professionals were ready for telemedicine implementation. To successfully implement telemedicine, it is crucial to prioritize capacity building, improve IT infrastructure, and involve more female healthcare professionals in the process.

## Background

Telemedicine is the delivery of healthcare services using information, communication, and technologies for patient diagnosis, treatment, and follow-up when distance is a main determinant factor for the delivery of care (1–3). It includes delivery of healthcare services, like consultation, follow-up and monitoring, and other healthcare services via voice calls, text messages, video calls, and other mechanisms for patients at their convenience (i.e., office, home, etc.) (4). Telemedicine is incredibly promising in improving healthcare quality, and making healthcare easily accessible for the rural community and underserved urban areas (2–5).

Different pilot and national telemedicine initiatives have been implemented in African countries. However, only 10% of health systems in Africa integrated telemedicine. Moreover, more than 75% of telemedicine projects worldwide failed without yielding significant improvement to the health system, the majority of them being from Africa (6, 7). Different factors are attributed to the lower rate of adoption of telemedicine. The factors have been categorized under users (patients and clients) factors, organizational factors, and staff and programmer factors. From the staff perspective, resistance to change, and lack of readiness are the most commonly cited reasons for the low adoption rate (7–9).

Studies showed that assessing and addressing readiness and factors associated with readiness before implementation is crucial for a telemedicine project's success. Readiness evaluation refers to the preparedness of healthcare systems and health professionals to adopt changes that have been brought about by the implementation of computerized systems (10). Health care provider's readiness is among the commonly cited critical factors for telemedicine project implementation success and sustainability (11).

Readiness of the health professionals is defined as the propensity of health workers to use telemedicine for providing care. It has 3 domains. This includes core readiness, engagement readiness, and structural readiness. Core readiness is defined as “The realization of needs and expressed dissatisfaction with the current way of working, such as inefficient documentation, patient privacy, and data confidentiality” (12). Engagement Readiness is defined as an active willingness to implement telemedicine and volunteer to receive telemedicine training. Structural readiness is concentrated on technical infrastructure and staff skills (13). Overall health providers’ readiness is determined at the intersection of core readiness, engagement readiness, and structural readiness (14).

Implementing telemedicine sustainably into the health system could have solved problems related to low access to health care, high patient load for health facilities, and high travel costs for patients and professionals in lower and middle-income countries (15). According to the WHO report, the Universal Health Coverage Index increased only by 3 index points from 2015 to 2019. Additionally, 4.5 billion people worldwide are still not covered with basic health services and out-of-pocket expenditure dragged 344 million people into extreme poverty. Globally 2 billion people still face financial hardship to access essential health services while the majority are in Africa (16).

Ethiopia under the national strategy of “Digital Ethiopia 2025: A Digital Strategy for Ethiopia Inclusive Prosperity”, started to implement different national initiatives including in the health sector. Ethiopia also issued a national electronic health (E-health) strategy in 2014. Moreover, Ethiopia developed a national digital health blueprint to guide the implementation of digital health technologies including telemedicine until the end of 2030 serving as a guideline (17). It identified remote care and telemedicine among the top ten priorities to be implemented by 2030. Since then, the Government of Ethiopia and its partners implemented telemedicine projects at selected hospitals mainly amid the COVID-19 pandemic. Moreover, During the period COVID-19 outbreak, Ethiopia issued national telehealth guidelines that gave direction on how to use telemedicine for pandemic control and routine health services delivery (14).

Different initiatives have been tried to increase awareness and readiness of health professionals towards digital health in Africa and Ethiopia. Incorporating health informatics in medical education, short-term capacity-building training, and training in health informatics at the undergraduate level are some of the tried initiatives (18).

Despite this, there is limited study conducted assessing the readiness of health professionals to implement telemedicine in Ethiopia and the study area in particular. Thus, this study aimed to assess the health professionals’ readiness to implement telemedicine and factors associated with readiness in Gamo zone public hospitals while addressing variables that were not included in previous studies.

## Methods

### Study design, area, and period

An institution-based, multi-center cross-sectional study was conducted from July 10 to August 10, 2024, in Gamo Zone Public Hospitals, South Ethiopia. Arba-Minch is the administrative town of the Gamo zone, and it is located 358 kilometers away from Addis Ababa. According to the Gamo Zone Health Department's 2024 report, Gamo Zone has a total population of 1,024,567, and of these, 560,056 are male. In general, in Gamo Zone, there are 5 primary hospitals, 1 general hospital, 1 private primary hospital, 60 health centers, 303 health posts, 38 private medium clinics, 61 private primary clinics, and 47 pharmacies. In general, in public health facilities, there are 1664 male and 1896 female health workers.

### Population

All health professionals working in Public hospitals in the Gamo zone were the source population. All selected health professionals working in public hospitals in the Gamo zone during the data collection period were the study population.

### Eligibility criteria

Health professionals having a minimum of six months of work experience were included. This is because they are familiar with the work environment and may have knowhow the application of telemedicine technology for healthcare delivery. The six-month experience threshold ensured familiarity with hospital workflows and potential exposure to digital health technologies. Those who were away from the office for training or annual leave were excluded from the study.

### Sample size determination

The sample size was determined using a single population proportion formula:

$$n=\frac{{\left(\text{Z}\frac{\alpha }{2}\right)}^{2}\text{p}\;(1-\text{p})}{{\text{d}}^{2}}$$

Where n: sample size Zά/2: 1.96 (using a 95% confidence interval and *α* = 0.05) £: margin of error (0.05), and 10% non-response rate. Since there is no previous study conducted in public hospitals in Ethiopia, 50% was taken to get the maximum possible sample size.

$$n=\frac{{\left(\text{Z}\frac{\alpha }{2}\right)}^{2}\text{p}\;(1-\text{p})}{{\text{d}}^{2}}=(1.96{)}^{2}0.5*0.5/(0.05*0.05)=384+38.4=422.8=423$$

The sample size for the second objective was also calculated using EPI-INFO software. However, it was less than the first objective. So, 423 was taken as the final sample size.

### Sampling procedure

There are six public hospitals in the Gamo zone. Five of them are primary hospitals and 1 general hospital. The total sum of health professionals in the six hospitals is 980. Each hospital received a proportionate share based on the number of medical personnel (data from the Gamo zone health department was taken for each hospital), and finally, the study participants from each hospital were selected using a simple random sampling technique (Supplementary Figure 1).

### Operational definition

Core readiness: “The realization of needs and expressed dissatisfaction with the current way of working, such as inefficient documentation, patient privacy, and data confidentiality” (12). A study participant who scored 50% or more for core readiness item (3 items) questions is assumed to have core readiness (19–21).

Engagement Readiness: “defined as an active willingness to implement telemedicine and volunteer to receive telemedicine training” (22). A study participant who scored 50% or more for engagement item questions (7 item questions) is assumed to have engagement readiness (19–21).

Structural readiness is concentrated on technical infrastructure and staff skills (13). A study participant who scored 50% or more for structural readiness item questions (7 item questions) is assumed to have structural readiness (19–21).

Overall health providers’ readiness is determined at the intersection of core readiness, engagement readiness, and structural readiness (14).

A health professional is said to have readiness if he or she has core readiness, engagement readiness, and structural readiness (14, 19, 20).

Knowledge about Telemedicine: the knowledge about telemedicine part of the questionnaire included 13 items. Respondents who scored 50% and above were considered to have adequate knowledge and below 50% were considered as having inadequate knowledge (19, 20, 23, 24).

Attitude towards Telemedicine: The attitude of the participant was assessed using 20 Likert item scales 1 denoting strongly disagree to 5 strongly agree for positively worded attitude questions. For negatively worded attitude questions reverse scoring was used (strongly disagree 5 to strongly agree 1). Participants who scored less than 50% and less, were considered as having unfavorable attitudes, and more than 50% were considered as having favorable attitudes (11, 25, 26).

### Data collection

The data was collected by using a self-administered structured standard questionnaire which was developed in English language from different literatures in Ethiopia in various e-health projects, and modified for telemedicine (10, 13, 14, 19, 21, 27, 28). The questionnaire included five parts. These were socio-demographic characteristics of health professionals, core readiness, engagement readiness, structural assessment, health professionals’ technical and behavioral related factors assessment, and finally organizational factors. The data was collected by six Bachelor of Science in Public Health professionals using the Kobo toolbox android application, and two Master of Public Health professionals were supervisors for data collection.

### Data quality

The data collectors and supervisors received one day of training on the extraction tool, ethical conduct, confidentiality, and software use to ensure consistency. A pre-test was performed on 5% of study participants at Karat Primary Hospital two weeks before the actual gathering of data, and the obtained data was reviewed for accuracy, completeness, clarity, and consistency. The reliability of items’ used for assessing readiness, knowledge, and attitude was checked using Cronbach's alpha and it was 0.94, 0.85, and 0.80, respectively showing higher reliability. The questionnaire was checked by three researchers of Health informatics for its face and content validity.

### Data processing and analysis

The collected data by using Kobo toolbox v2024.1.3 was exported to SPSS version 26 for data processing and analysis. To summarize continuous data, the mean and standard deviation were employed, as frequency distribution for categorical data. The result was presented in texts, graphs, and tables. The variance inflation factor (VIF) was below 1 for all variables, indicating no multicollinearity. The Hosmer-Lemeshow test (*p* = 0.3) indicated a good model fit for logistic regression. Both bivariable and multivariable logistic regression analysis was used to identify associated factors. Variables with *p*-values less than or equal to 0.25 were taken to multivariable logistic regression to control for possible confounding effects of independent variables. Variables with *p*-values less than 0.05 at a 95% confidence interval and adjusted odd ratios were considered significantly associated with readiness for telemedicine implementation.

### Ethical clearance

Ethical approval was obtained from the Institutional Review Board of Arba Minch University, College of Medicine and Health Science. A letter of support for hospital administrators was written by the School of Public Health. Permission letters were obtained to interview the healthcare professionals. All concerned bodies of the research area were notified and consent was taken. The participants were briefly told about the research process and purpose. Informed oral consent was taken from participants before the interview process. All methods applied in this research were per relevant guidelines, principles, and ethical standards of the Declaration of Helsinki.

## Result

### Socio-demographic characteristics of respondents

A total of 417 healthcare professionals in six hospitals participated in this study with a 98.6% response rate. Among the total respondents, 223(53.5%) of the respondents were male. The age of respondents ranged from 25 to 46, while, 179 (42.9%) of respondents fell in the range of 25–29 years, and the mean age was 31.78 years with a standard deviation of 5.31 years. The majority (63.3%) of respondents were married. In terms of profession, 143 (34.3%) of respondents were nurses and 5 (1.2%) of them were Integrated Emergency Surgical Officers (IESO). Regarding educational level, 273 (65.5%) of them were first-degree holders and 26 (6.2%) of them were master's degree holders (Table 1).

**Table 1:** Socio-demographic characteristics of respondents in the study of assessment of healthcare professionals’ readiness to implement telemedicine and associated factors among healthcare professionals working in public hospitals in Gamo zone, south Ethiopia, 2024(*N* = 417).

Variables	Categories	Frequency (*n*)	Percentage (%)
Sex	Male	223	53.5
	Female	194	46.
Age	25–29	179	42.9
	30–34	106	25.4
	35 and above	132	31.7
Marital status	Single	147	35.3
	Married	264	63.3
	Others^a^	6	1.4
Profession	Nurse	143	34.3
	Health officer	63	15.1
	General practitioner	39	9.4
	Midwifery	49	11.8
	Medical laboratory	43	10.3
	Pharmacist	43	10.3
	Specialist	10	2.4
	Radiographer	7	1.7
	Anesthetist	7	1.7
	IESO	5	1.2
Salary	< 89 USD	18	4.3
	89–178 USD	353	84.7
	> 179 USD	46	11
Education	Diploma	118	28.3
	Degree	273	65.5
	Masters	26	6.2
Work experience	Less than 2 years	87	20.9
	2–3 years	53	12.7
	4–5 years	38	9.1
	5 years and above	239	57.3

Other^a^, divorced and widowed IESO, Integrated emergency surgical officer; USD, United States Dollar. 1USD = 56 ETB.

### Healthcare professionals’ readiness to implement telemedicine services

Health professionals’ core, engagement, and structural readiness to implement telemedicine systems was found to be 298(71.5%), 293(70.3%), and 291(69.3%) respectively (Table 2). The Overall health providers’ readiness was found to be 287(68.8%) (95% CI: 64.3%–73%). (Supplementary Figure 2).

**Table 2:** Core, engagement, and structural readiness for the study of assessment of health providers’ readiness and associated factors working in Gamo zone public hospitals, 2024 (*N* = 417).

Readiness questions	Completely disagree *N* (%)	Disagree *N* (%)	Neutral *N* (%)	Agree *N* (%)	Completely agree *N* (%)
Core readiness					
I am dissatisfied with the current ways of patient management/follow-up and Treatments.	104 (24.9)	136 (32.6)	70 (16.8)	83 (19.9)	24 (5.8)
Patients are having difficulty getting the medical care they need because providers are too far away or lack transportation.	12 (2.9)	105 (25.2)	41 (9.8)	237 (56.8)	22 (5.3)
I need to address/solve patient problems that could be met by Telemedicine	21 (5)	84 (20.1)	32 (7.7)	34 (8.2)	246 (59.8)
Engagement readiness					
I think I will be the first one to use technologies for patient management.	12 (2.9)	131 (31.4)	69 (16.5)	152 (36.5)	53 (12.7)
I believe the use of Telemedicine health services technologies could improve patient management/care.	13 (3.1)	100 (24)	64 (15.3)	210 (50.4)	30 (7.2)
I am willing to work with the ICT team	13 (3.1)	71 (17.3)	57 (13.7)	232 (55.6)	43 (10.3)
I need to interact with other practitioners.	9 (2.2)	79 (18.9)	52 (12.5)	241 (57.8)	36 (8.6)
I have examples of technologies that are used to support or monitor patients.	29 (7)	72 (17.3)	53 (12.7)	238 (57.1)	25 (6)
I will communicate with other practitioners/public about the benefits of telemedicine.	25 (6)	94 (22.5)	61 (14.6)	204 (48.9)	33 (7.9)
I am willing to make the initial additional/extra investment in time	25 (6)	79 (18.9)	96 (23)	187 (44.8)	30 (7.)
Structural readiness					
Believes Telemedicine health services can address scheduling and workload concerns.	30 (7.2)	64 (15.3)	62 (14.9)	207 (49.6)	54 (54)
I have 24-hour access to Telemedicine health services equipment (internet, computer, smartphones, and others).	29 (7)	160 (38.4)	60 (14.4)	149 (35.7)	19 (4.6)
I have a plan to use technologies for patient management	25 (6)	139 (33.3)	58 (13.9)	160 (38.4)	35 (8.4)
I believe in the reliability/trustworthiness of Telemedicine health services equipment and have good technical support.	31 (7.4)	139 (33.3)	93 (22.3)	142 (34.1)	12 (2.9)
I have access/contact to a well-known clinical consultation network (group).	38 (9.1)	142 (34.1)	113 (27.)	106 (25.4)	18 (4.3)
I am provided with reliable clinical content and continuous medical education (CME) through telemedicine	23 (5.5)	78 (18.7)	64 (15.3)	230 (55.2)	22 (5.3)
I attend to issues regarding liability and licensing when using telemedicine	13(3.1)	71(17)	117(28)	190(45.6)	26(6.2)

### Health professionals’ behavioral and technical factors

Among the total respondents, 266 (63.8%) had adequate knowledge about telemedicine. Among the total respondents, 283 (67.9%) heard about telemedicine before. Among those who heard about telemedicine before, 141 (49.8%) of them got the information from the Internet and only 12 (4.2%) of them heard about telemedicine from training (Table 2). Similarly, 251 (61.9%) of respondents had a favorable attitude towards telemedicine (Tables 3, 4).

**Table 3:** Knowledge questions study of assessment of healthcare professional's readiness to implement telemedicine and associated factors among healthcare professionals working in public hospitals in Gamo zone, South-Ethiopia, 2024 (*N* = 417).

Questions	Yes *N* (%)	No *N* (%)
Have you ever heard about telemedicine before?	283 (67.9)	134 (32.1)
If yes to number 1, What was your source of information? (*N* = 283)	Options	*N* (%)
	School	51 (18.2)
	Internet	141 (49.8)
	Mass media	79 (27.9)
	Training	12 (4.2)
I know on at least one telemedicine application type (video, audio, text, M-health,).	211 (50.6)	206 (49.4)
I have an awareness of E-health types like EMR, tele monitoring, personal health records, and wearables.	130 (31.2)	287 (68.8)
Telemedicine improves the quality of healthcare delivery.	344 (82.5)	73 (17.5)
Telemedicine reduces overload and hospital overcrowding.	366 (87.7)	51 (12.2)
Telemedicine reduces transportation costs due to unnecessary appointments that could be done at remote	375 (89.9)	42 (10.1)
Telemedicine reduces patients’ time to get care.	318 (76.3)	99 (23.7)
Telemedicine reduces clinician time to deliver care	317 (76)	100 (24)
Did you know about any Telemedicine guidelines (guidelines of Ethiopia for telemedicine or any other guideline)?	22 (5.3)	395 (94.7)
Does Telemedicine reduce the gap between the availability of health services and the demand for them?	246 (59)	171 (41)
Did you know about Telemedicine infrastructures?	187 (44.8)	230 (55.2)
Have you ever seen a Telemedicine process previously?	73(17.5)	344(82.5)

**Table 4:** Attitude questions study of assessment of health care professional's readiness and associated factors to implement telemedicine among healthcare professionals working in public hospitals in Gamo zone, South-Ethiopia, 2024 (*N* = 417).

Attitude questions	Completely disagree *N* (%)	Disagree *N* (%)	Neutral *N* (%)	Agree *N* (%)	Completely agree *N* (%)
I believe that telemedicine					
Reduces medical errors.	65 (15.6)	149 (35.7)	76 (18.2)	110 (26.4)	17 (4.1)
Helps health professionals perform their duties.	34 (8.2)	136 (32.6)	106 (25.4)	119 (28.5)	22 (5.3)
Enhance communication between providers.	75 (18)	213 (51.1)	44 (10.6)	83 (19.9)	2 (0.5)
Facilitates information dissemination for patients.	45 (10.8)	137 (32.9)	64 (15.3)	156 (37.3)	15 (3.6)
Increase the service delivery speed.	15 (3.6)	174 (41.7)	91 (21.8)	127 (30.5)	10 (2.4)
Increase timely access to information.	51 (12.2)	191 (45.8)	73 (17.5)	83 (19.9)	19 (4.6)
Enhances quality of healthcare	18 (4.3)	172 (41.2)	46 (11)	149 (35.9)	32 (7.7)
Enhances clinical decision	29 (7)	129 (30.9)	119 (28.5)	116 (27.8)	24 (5.8)
Improves communication between work units	10 (2.4)	253 (60.7)	45 (10.8)	109 (26.1)	0 (0)
Helps to provide all services needed easily	6 (1.4)	224 (53.7)	52 (12.5)	132 (31.7)	3 (0.7)
Helps to easily track patient records.	36 (8.6)	195 (46.8)	56 (13.4)	124 (29.4)	6 (1.4)
Easily shows the weakness in the health system.	56 (13.4)	149 (35.7)	56 (13.4)	138 (33.1)	18 (4.3)
Threaten staff positions	31 (7.4)	137 (32.9)	113 (27.1)	124 (29.7)	12 (2.9)
Creates new responsibility for staff	32 (7.7)	215 (51.6)	43 (10.3)	113 (27.1)	14 (3.4)
Reduces workload	9 (2.2)	83 (19.9)	73 (17.5)	236 (56.6)	16 (3.8)
Improves clinical work	69 (16.5)	202 (48.4)	47 (11.3)	90 (21.6)	9 (2.2)
Telemedicine Increase staff workload	65 (15.6)	154 (36.9)	48 (11.5)	139 (33.3)	11 (2.6)
Threatens patient confidentiality and privacy	55 (13.2)	233 (55.9)	72 (17.3)	39 (9.4)	18 (4.3)
Increases cost	28 (6.7)	237 (56.8)	46 (11)	94(22.5)	12(2.9)
Increases legal issues	39(9.4)	205(49.2)	44(10.6)	112(26.9)	17(4.1)

In this study, 344(82.5%) reported that they believed they had basic computer skills and 346(83%) of the respondents reported that they were computer literate. Additionally, 224 (53.7%) had personal computers (Table 5).

**Table 5:** Behavioral and technical factors in the study of assessment of healthcare professional's readiness to implement telemedicine and associated factors among healthcare professionals working in public hospitals in Gamo zone, South-Ethiopia, 2024 (*N* = 417).

Variable	Category	Frequency (*n*)	Percentage (%)
Knowledge	Adequate knowledge	266	63.8
	Inadequate knowledge	151	36.2
Attitude	Favorable attitude	258	61.9
	Unfavorable attitude	159	38.1
Believe in your basic computer skill	Yes	344	82.5
	No	73	17.5
Believe in own computer literate.	Yes	346	83
	No	71	17
Computer related training	Yes	58	13.9
	No	359	86.1
Having personal computer	Yes	224	53.7
	No	193	46.7
Internet use for health information access	Yes	345	82.7
	No	72	17.3
Comfort while using the computer	Yes	272	65.22
	No	145	34.78
E-health experience before	Yes	96	23.02
	No	321	76.98
Have you taken training on E-health Before (EMR, telemedicine, etc.,)	Yes	12	2.88
	No	405	97.12

### Organizational related factors

Out of the total respondents, 187(44.8%) had computer access at their workplace and only 126(30.2%) of respondents had internet access at their workplace (Table 6).

**Table 6:** Organizational factors in the study of assessment of healthcare professionals’ readiness to implement telemedicine and associated factors among healthcare professionals working in public hospitals in Gamo zone, South-Ethiopia, 2024 (*N* = 417).

Variable	Category	Frequency (*n*)	Percentage (%)
Computer access at the workplace	Yes	187	44.85
	No	230	55.15
Internet access at the workplace	Yes	126	30.22
	No	291	69.78
Available IT support	Yes	197	47.24
	No	220	52.76
Having a backup power generator	Yes	315	75.7
	No	102	24.3
Plan to implement E-health at the hospital	Yes	37	8.87
	No	380	91.13

### Factors associated with healthcare professionals’ readiness to implement telemedicine services

In bivariable logistic regression analysis sex of participants, educational level, work experience, knowledge, attitude, internet use for health information access, computer access at the workplace, Internet access at the workplace, having basic computer skills, and having information on a plan to implement E-health at the hospital level were associated with readiness to implement telemedicine services at the *p*-value of ≤0.25. In multivariable logistic regression analysis, the sex of participants, knowledge, attitude, and computer access at the workplace were found significantly associated with readiness to implement telemedicine at a *P*-value of <0.05.

Female healthcare professionals were 1.78 times more likely to be ready to implement telemedicine compared to male healthcare professionals (95% CI: 1.12–2.84). Health professionals with adequate knowledge were 2.1 times more likely to be ready to implement telemedicine services than those with inadequate knowledge (95% CI: 1.29–3.41). Additionally, Health professionals with favorable attitudes were 1.96 times more likely to be ready than those with unfavorable attitudes (95% CI: 1.28–3.13). Health professionals who had access to a computer at the workplace were 2.62 times more likely to be ready to implement telemedicine services than those who had no access to a computer at the workplace (95% CI: 1.26–3.93) (Table 7).

**Table 7:** Bivariate and multivariate analysis of the study of assessment of health care professional's readiness to implement telemedicine and associated factors to implement telemedicine among healthcare professionals working in public hospitals in Gamo zone, South-Ethiopia, 2024 (*N* = 417).

Variables	Categories	Outcome		COR (95%CI)	AOR (95%CI)	*p*-value
		Ready	Not ready			
Sex	Male	141	82	1	1	
	Female	146	48	1.78 (1.16–2.71)	1.78 (1.12–2.80)	**0** **.** **015**
Education	Diploma	74	44	1	1	
	Degree	193	80	1.4 (0.9–2.26)	1.23 (0.76–2.11)	0.38
	Masters	20	6	1.98 (0.74–5.31)	1.53 (0.53–4.41)	0.43
Work experience	< 2 years	57	30	1	1	
	2–3 years	38	15	1.33 (0.63–2.80)	1.35 (0.61–3.01)	0.46
	4–5 years	33	5	3.47 (1.23–9.8)	2.77 (0.91–8.32)	0.07
	> 5 years	159	80	1.05 (0.6–1.7)	1.01 (0.57–1.80)	0.97
Knowledge	Inadequate	87	67	1	1	
	Adequate	203	63	2.76 (1.68–3.94)	2.10 (1.29–3.41)	**0**.**003**
Attitude	Unfavorable	99	60	1	1	
	Favorable	188	70	1.63 (1.01–2.45)	1.96 (1.23–3.131)	**0**.**005**
Basic computer skill	Yes	244	100	1.71 (1.02–2.87)	1.37 (0.69–2.72)	0.35
	No	43	30	1	1	
Internet use	Yes	245	100	1.75 (1.03–2.96)	0.91 (0.45–1.76)	0.74
	No	42	30	1	1	
Computer access	Yes	149	38	2.62 (1.68–4.02)	2.23 (1.26–3.93)	**0**.**005**
	No	138	92	1	1	
Internet access	Yes	96	30	1.68 (1.04–2.70)	1.01 (0.55–1.84)	0.98
	No	191	100	1	1	
Plan to implement e-health	Yes	22	15	0.63 (0.32–1.27)	0.63 (0.29–1.35)	0.24
	No	265	115	1	1	

AOR, adjusted odd ratio COR: crude odds ratio CI: confidence interval 1: reference.

Significant *p*-value < 0.05 were in bold.

## Discussion

This study assessed the health professional's readiness to implement telemedicine services and associated factors at six public hospitals in the Gamo zone. It was found that overall healthcare professionals’ readiness for telemedicine services implementation was 68.8%. This finding shows that the majority of health professionals are ready for telemedicine implementation. Therefore, hospitals should design and implement telemedicine systems after doing thorough research on which segment of service delivery point highly benefits from telemedicine.

The findings of this study were consistent with those studies conducted in Amhara region private hospitals (65.4%) (14), Ugandan physicians(64.7%) (26), Mauritius (64.5%) (29), and palliative nursing specialists in China (65%) (30).

However, the findings of this study were higher than the findings from a study conducted in China (50.1%) (31), Austria (58.2%) (32), and Nigeria (33%) (33). The possible reason for this discrepancy could be due to differences in data collection method, setting, period, and measurement differences. For instance, the study conducted in China among nurses used a convenience sampling method which might have caused sampling bias as it only relies on simply accessibility of participants limiting its representativeness of the broader population. This in turn could have resulted lower reported readiness level. Moreover, the study conducted in Austria (32) was conducted during pre-Covid-19. During COVID-19 awareness and utilization of telemedicine highly increased. This might explain the higher rate of readiness level in our setting. Additionally, it only included 41 participants which was much lower than our sample size.

On the contrary, the findings of this study were lower than the findings of a study from Ethiopia (77%) (13) and Uganda (76%) (26). The possible reasons for this discrepancy could be due to differences in study participants, infrastructure, and socio-demographic differences.

In this study, female health professionals were 1.78 times more likely to be ready than males to use telemedicine. This study was supported by studies conducted in Germany (34) and Ontario (35). This might be due to the perception that telemedicine could offer flexibility for work and this could lead to work-life balance and make them fulfill their caregiving role at home (family responsibility). Trends seen amid the COVID-19 outbreak and afterward, female physicians were active users of telemedicine. Moreover, female professionals have a strong focus on patient-centered care which might influence them to use virtual consultation for smooth working conditions (35). Therefore, when planning for telemedicine system implementation, hospitals should involve females as change agents as females were more ready to implement telemedicine systems in this research.

Healthcare professionals with adequate knowledge about telemedicine were 2.1 times more likely to be ready to use telemedicine than those with inadequate knowledge (95% CI: 1.29–3.41). This study was supported by studies conducted in Ethiopia (13, 14), Mauritius (29), Ghana (36), Uganda (9), and Saudi Arabia (25). This might be due to professionals having adequate knowledge about telemedicine and having a clear understanding of possible benefits, functionality, and potential for improving healthcare access. This in turn might have resulted in increased readiness for the adoption of telemedicine. Having adequate knowledge not only reduces uncertainty but also increases confidence in how to utilize telemedicine for the improvement of the health of the community. This shows that capacity-building activities should be given to health professionals to increase their awareness and raise their readiness level.

Respondents who had favorable attitudes towards telemedicine were 1.96 times more likely to be ready to implement telemedicine than those with unfavorable attitudes. This finding was supported with studies from Amhara region (14), Addis Ababa (13), Uganda (26), Ghana, Austria (32), and Mauritius (29). This might be due to having a positive perception of telemedicine and a belief in telemedicine that it could result in a positive impact on coverage of health care might drive readiness for implementation.

Among organizational factors, respondents who had computer access at the workplace were 2.23 times more ready than those who had no computer access at the workplace. This was supported by studies conducted in Ethiopia (14), Mauritius (29), and Lebanon (37). The possible reason could be professionals who had computer access at the workplace might get the chance to continuously expose themselves to computer and digital tools to support patients. This in turn might lead to higher readiness than those who had no access to a computer. As a result, it is strongly recommended that hospitals give priority to fulfillment information technology infrastructures in the workplace.

### Limitations of the study

This study focused on health professionals’ readiness for telemedicine implementation but did not assess organizational readiness, which encompasses infrastructure, policies, and training support that may influence professionals’ preparedness. This limitation restricts insights into systemic barriers, warranting future research on organizational factors to ensure comprehensive digital health adoption. Moreover, because the study was cross-sectional, it doesn’t infer causality. Additionally, it did not triangulate findings with qualitative studies due to feasibility issues which limited in-depth exploration of readiness barriers. Future studies should assess organizational readiness, employ longitudinal designs to track readiness trends, and use mixed-methods approaches to explore barriers qualitatively.

## Conclusion

This study found that around two-thirds of health professionals were ready for telemedicine implementation. Being female, having adequate knowledge, having a favorable attitude towards telemedicine, and having access to a computer at the office were significantly associated with the health professionals’ readiness to implement telemedicine. A majority of health professionals are for telemedicine implementation, the hospitals should implement telemedicine systems. To successfully implement telemedicine, it is crucial to prioritize capacity building, improve IT infrastructure, and involve more female healthcare professionals in the process. It would be better for future researchers if they could assess the organizational and patient readiness for telemedicine implementation. Additionally, they should use qualitative studies to explore the concerns of health professionals.

## Data Availability

The original contributions presented in the study are included in the article/Supplementary Material, further inquiries can be directed to the corresponding author.
