# Supplementary figures and images for: Readiness of health professionals for telemedicine implementation: multi-centered cross-sectional study in public hospitals, South Ethiopia
Source: Front Digit Health. 2025 Jul 2;7:1554199. doi: 10.3389/fdgth.2025.1554199 (PMC12263920; doi:10.3389/fdgth.2025.1554199)

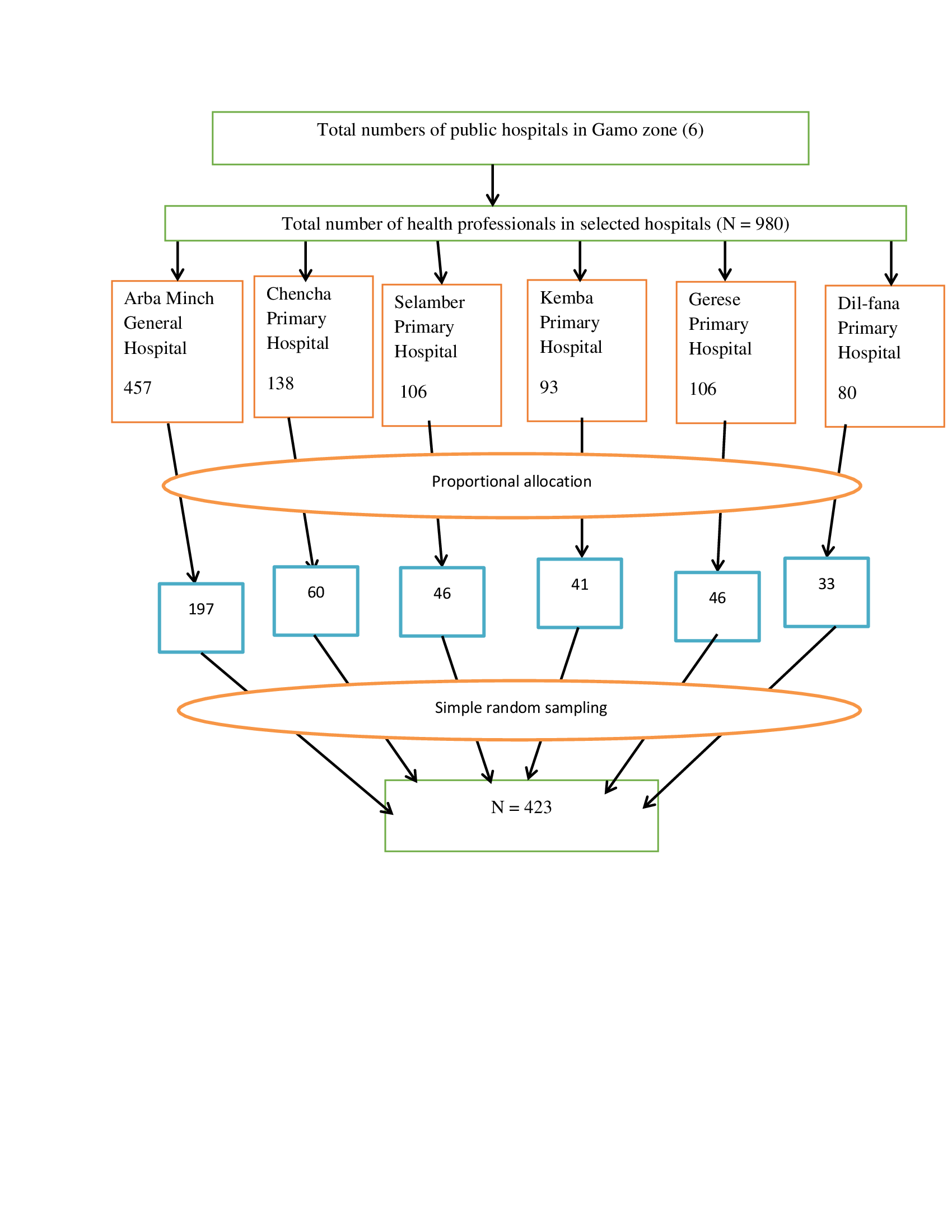

Supplement: Supplementary file 1 [file Datasheet1.zip › Figures suplemntary data/Figure 1.tiff]
